# Development and safety of PI3K inhibitors in cancer

**DOI:** 10.1007/s00204-023-03440-4

**Published:** 2023-02-11

**Authors:** Miaomiao Yu, Jiajia Chen, Zhifei Xu, Bo Yang, Qiaojun He, Peihua Luo, Hao Yan, Xiaochun Yang

**Affiliations:** 1 Center for Drug Safety Evaluation and Research of Zhejiang University, College of Pharmaceutical Sciences, Zhejiang University, 866 Yuhangtang Road, Zijingang Campus, Hangzhou, 310058 Zhejiang People’s Republic of China; 2 Institute of Pharmacology & Toxicology, College of Pharmaceutical Sciences, Zhejiang University, Hangzhou, 310058 Zhejiang People’s Republic of China; 3 Hangzhou Institute of Innovative Medicine, College of Pharmaceutical Sciences, Zhejiang University, Hangzhou, 310058 People’s Republic of China; 4 Innovation Institute for Artificial Intelligence in Medicine, Zhejiang University, Hangzhou, 310018 Zhejiang People’s Republic of China

**Keywords:** PI3K, Inhibitors, Toxicity, Management, Mechanism

## Abstract

The phosphatidylinositol 3-kinase (PI3K) signalling pathway regulates cell survival, proliferation, migration, metabolism and other vital cellular life processes. In addition, activation of the PI3K signalling pathway is important for cancer development. As a result, a variety of PI3K inhibitors have been clinically developed to treat malignancies. Although several PI3K inhibitors have received approval from the Food and Drug Administration (FDA) for significant antitumour activity, frequent and severe adverse effects have greatly limited their clinical application. These toxicities are mostly on-target and immune-mediated; nevertheless, the underlying mechanisms are still unclear. Current management usually involves intervention through symptomatic treatment, with discontinuation if toxicity persists. Therefore, it is necessary to comprehensively understand these adverse events and ensure the clinical safety application of PI3K inhibitors by establishing the most effective management guidelines, appropriate intermittent dosing regimens and new combination administration. Here, the focus is on the development of PI3K inhibitors in cancer therapy, with particular emphasis on isoform-specific PI3K inhibitors. The most common adverse effects of PI3K inhibitors are also covered, as well as potential mechanisms and management approaches.

## Introduction

Excessive activation of the phosphatidylinositol 3-kinase (PI3K) signalling pathway is considered to be one of the hallmarks of human malignancies (Fruman et al. 2017). The pathway is activated through diverse genomic alterations, including the oncogenes phosphatidylinositol-4,5-bisphosphate 3-kinase catalytic subunit alpha (*PIK3CA*) and phosphoinositide-3-kinase regulatory subunit 1 (*PIK3R1*), the tumour suppressor gene phosphatase and tensin homolog (*PTEN*), and other crucial genes, which hold promise as effective therapeutic targets (Janku et al. 2018). In addition, the PI3K axis plays a fundamental role in the survival, cell proliferation, metabolism and inflammation of several cellular processes (De Santis et al. 2019). Hence, it follows that the significance of the PI3K pathway has facilitated pharmacological intervention targeting PI3K.

**Fig. 1:**
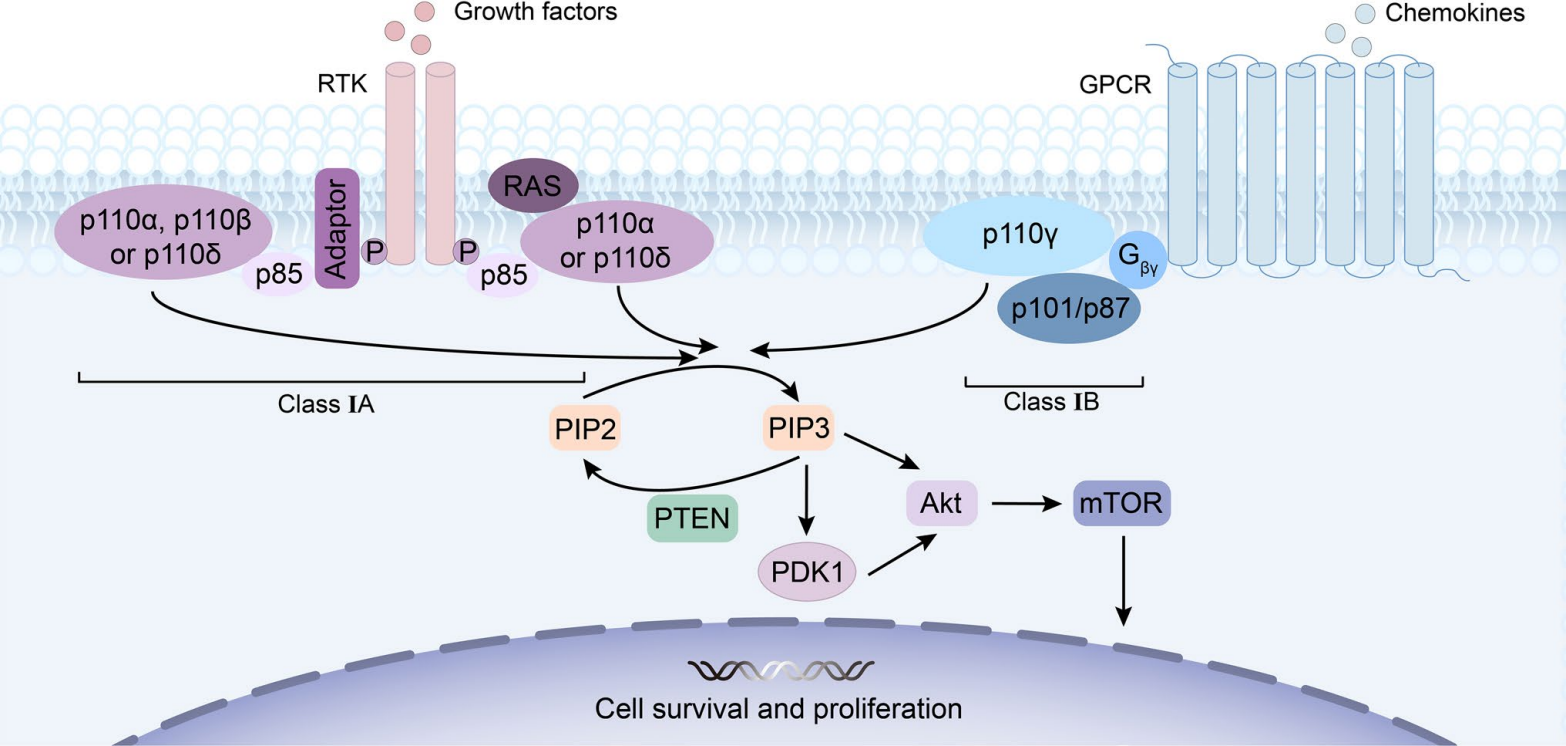
Overview of the phosphatidylinositol 3-kinase (PI3K) signalling pathway. The PI3K signalling pathway is activated by G protein-coupled receptor (GPCR) or receptor tyrosine kinase (RTK). Class I PI3Ks activate phosphatidylinositol 4,5‑bisphosphate (PIP2) to generate phosphatidylinositol 3,4,5‑trisphosphate (PIP3), and PIP3 can be dephosphorylated by phosphatase and tensin homolog (PTEN) to form PIP2. PIP3 further induces the activation of the downstream protein kinases phosphoinositide-dependent kinase 1 (PDK1), protein kinase B (Akt), and mammalian target of rapamycin (mTOR) to regulate cell survival and proliferation. Class I PI3Ks are divided into class A and class B. The class A PI3Ks are further subdivided into catalytic subunit p110α, p110β, p110δ with p85 regulatory subunit, and class IB include p110γ with p87 or p101 regulatory subunit

PI3K is a family of lipid kinases that have been divided into three classes (I, II, III) based on sequence homology and substrate specificity (Miller et al. 2019). There are few reports on the specific functions of class II and class III PI3Ks relative to class I PI3Ks. Class II PI3Ks consist of the p110-like catalytic subunit and are capable of regulating the internalization of receptors. Class III PI3Ks are key proteins for vesicle transport from the Golgi to the vacuole in budding yeast (Aksoy et al. 2018). Class I PI3Ks are closely related to cancer and have been studied in-depth. Focus is on the class I PI3Ks, a heterodimer composed of a regulatory subunit and a catalytic subunit, which are divided into class IA and class IB due to different coupled receptors. Class IA is activated by growth factor receptor tyrosine kinase (RTK) and class IB is activated by G-protein-coupled receptor (GPCR) (Fig. 1). Class IA PI3Ks are further subdivided into p110α, p110β, p110δ with regulatory subunit p85, and class IB only includes p110γ with regulatory subunit p87 or p101 (Bilanges et al. 2019). Isoform-specific functions are related to the expression levels in different tissues. p110α and p110β are widely distributed throughout tissues, while p110δ is highly expressed in haematopoietic cells, and p110γ is mainly expressed in leukocytes (Thorpe et al. 2015). Using gene targeting studies, p110α was identified as the crucial isoform involved in vascular remodelling (Vantler et al. 2015), and p110β was considered to play a major role in platelet physiology (Moore et al. 2019); p110δ and p110γ regulate diverse aspects of the function of T and B lymphocytes (De Henau et al. 2016; Horwitz et al. 2018). Therefore, the drug’s effectiveness may be increased by specific PI3K subtype inhibition, but this could also result in more severe adverse events. (Esposito et al. 2019).

PI3K inhibitors have shown desired therapeutic effects in various cancer treatments. Among these PI3K inhibitors, copanlisib, alpelisib, idelalisib, duvelisib and umbralisib have been approved by the Food and Drug Administration (FDA), although approvals/accelerated application indications of partial inhibitors (idelalisib, duvelisib and umbralisib) have been withdrawn (Meng et al. 2021). In addition, several PI3K inhibitors are undergoing clinical trials. Despite ongoing research on this target, unintended side effects such as hyperglycaemia, rash, diarrhoea/colitis, hepatotoxicity and hypertension continue to be a major barrier to the development of PI3K inhibitors (Hanker et al. 2019).

In this review, we provide a comprehensive summary of the PI3K inhibitors that have been approved or in clinical trials (Table 1). Representative adverse effects associated with PI3K inhibitors, management guidelines and the conceivable mechanisms that have been reported are also discussed.

**Table 1:**
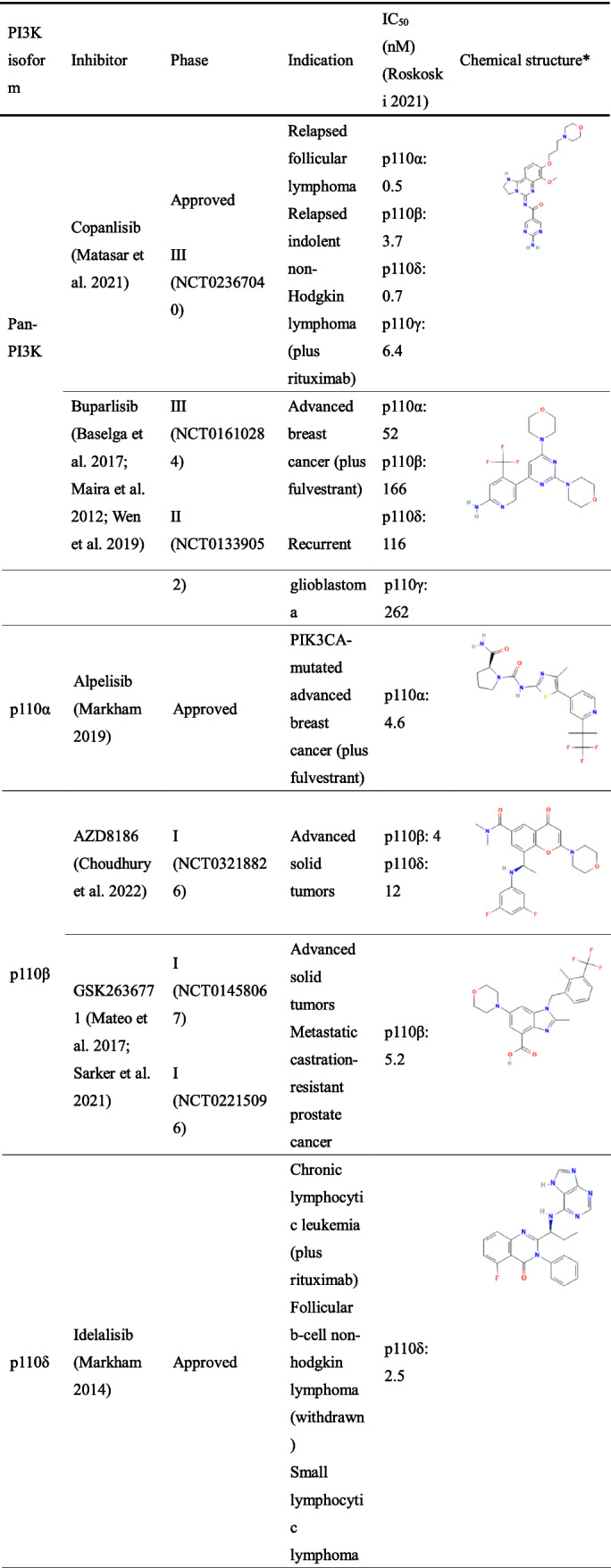

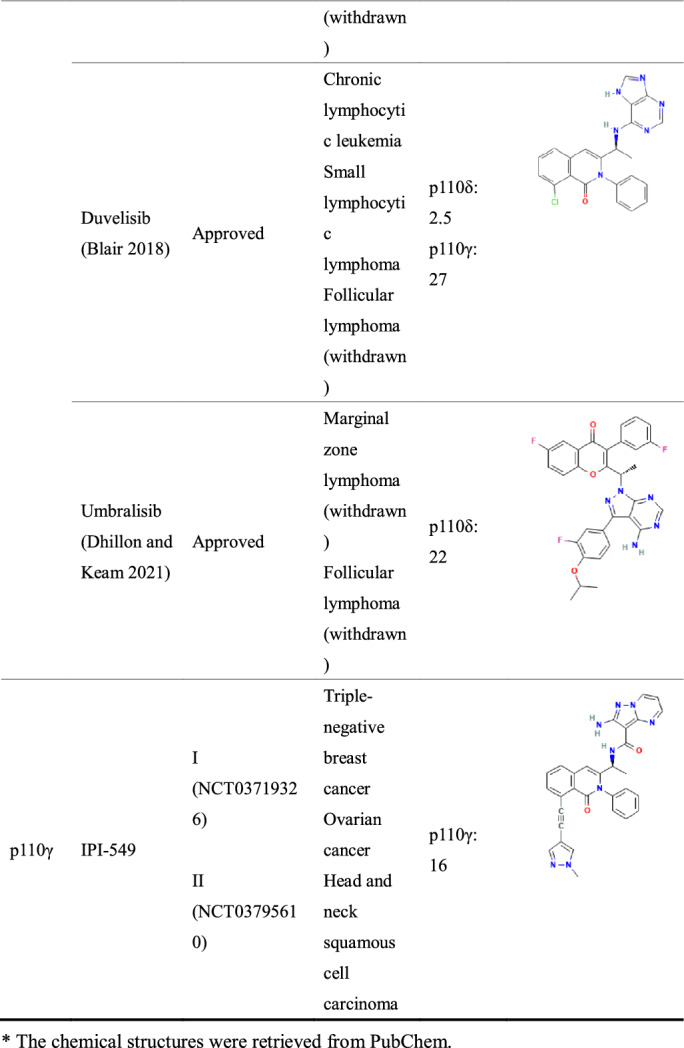
The typical and clinically developed PI3K inhibitors.

## The typical PI3K inhibitors approved or in clinical trials

### Pan-PI3K inhibitors

Due to the lack of a thorough understanding of the structure of the PI3K protein and isoforms in the early stage, PI3K inhibitors targeting the four isoforms of class I PI3K were mainly developed. In addition to playing different roles in tumour proliferation, various isoforms are often associated with multiple physiological functions, such as glucose metabolism, inflammation and immunity. Thus, pan-PI3K inhibitors have inevitably increased safety risks. In particular, the high incidence of metabolic-related adverse events, such as hyperglycaemia, has led to limited clinical doses (De Santis et al. 2019; Janku et al. 2018).

### Copanlisib

Copanlisib (Aliqopa^™^; BAY 80–6946; Bayer AG) is an intravenous pan-class I PI3K inhibitor with potent activity against all four isoforms (Markham 2017) and was approved by the FDA in May 2017 for the treatment of adult patients with relapsed follicular lymphoma (FL) who have received at least two prior systemic therapies. In addition, copanlisib shows greater efficacy and safety both as monotherapy and in combination in various clinical trials. In a phase II study, copanlisib as a single agent also demonstrated significant efficacy with relapsed or refractory indolent lymphoma (NCT01660451) (Dreyling et al. 2017b) and relapsed or refractory diffuse large B cell lymphoma (NCT02391116) (Lenz et al. 2020), and the median progression-free survival (PFS) was 11.2 months and 2.4 months, respectively. Copanlisib plus gemcitabine act synergistically when treating peripheral T cell lymphomas (NCT03052933); PFS was 6.9 months, and median overall survival (OS) was not reached (Yhim et al. 2021). In addition, copanlisib combined with rituximab improved PFS (21.5 months versus 13.8 months in the placebo plus rituximab group) in patients with relapsed indolent non-Hodgkin lymphoma in the CHRONOS-3 study (NCT02367040) (Matasar et al. 2021). Hyperglycaemia, diarrhoea and hypertension are the most frequent adverse events with copanlisib, but all of them are minor (Dreyling et al. 2020). Notably, although other PI3K inhibitors have remarkable safety concerns, copanlisib has a low incidence of severe toxicities may be due to the intravenous route of administration and intermittent dosing schedule (Killock 2021; Munoz et al. 2021).

### Buparlisib

Buparlisib (NVP-BKM120; Novartis) is an oral pan-PI3K inhibitor that selectively inhibits all isoforms of class I PI3K (Maira et al. 2012). On one hand, buparlisib has shown improved PFS in the clinical development of combined treatment, but on the other hand, it is limited by extensive toxicities (van Dam 2019). The BELLE-2 (NCT01610284) and BELLE-3 (NCT01633060) trials reported that buparlisib plus fulvestrant was effective in hormone receptor (HR)-positive, human epidermal growth factor receptor 2 (HER2)-negative, advanced breast cancer, with a median PFS of 6.9 months and 3.9 months, respectively. However, the toxicities associated with this combination, such as increased alanine aminotransferase (ALT) and aspartate aminotransferase (AST), hyperglycaemia and rash, led to clinical trial discontinuation (Baselga et al. 2017; Di Leo et al. 2018). Other studies have shown that buparlisib failed to have sufficient antitumour activity as a single agent or even plus carboplatin/lomustine in patients with recurrent glioblastoma (Rosenthal et al. 2020; Wen et al. 2019). Although some clinical trials suggest that buparlisib showed modest activity in solid tumours, this may be due to the different tissue distributions and functions of the various subunits. To increase efficacy while reducing the toxicity of PI3K inhibitors, future development of PI3K inhibitors should concentrate on isoform-selective PI3K inhibitors (McPherson et al. 2020; Soulieres et al. 2017).

### Isoform-specific PI3K inhibitors

The four isoforms of class I PI3Ks (α, β, δ, γ) have different tissue distributions and physiological functions, which affect the inhibitory effect of isoform-specific inhibitors on various tumours and the incidence of adverse effects (Table 2).

**Table 2:** Catalytic subunit typing of class I PI3K and their main physiological functions

Catalytic subunit	p110α (Zhang et al. 2021)	p110β (Moore et al. 2019)	p110δ (Xenou and Papakonstanti 2020)	p110γ (Nurnberg and Beer-Hammer 2019)
Tissue expression	Widely expressed	Widely expressed	Mainly expressed in hematopoietic cells	Mainly expressed in leukocytes
Physiological function	Insulin signalling; glucose metabolism; angiogenesis; PIK3CA-mutated in solid tumor;	Insulin signalling; glucose metabolism; platelet function;	B cell, T cell and mast cell development and function; antigen-dependent responses;	Driving leukocyte chemotaxis and recruitment; -adrenergic receptor (β-AR) signalling;

### PI3Kα inhibitors

The *PIK3CA* gene encodes the p110α catalytic subunit of PI3K, and its mutation occurs at a high rate in endometrial, breast, bladder, cervical and colorectal cancers (Arafeh and Samuels 2019). The *PIK3CA* activation mutation is among the most common oncogenic mutations described in breast cancer to date (Arafeh and Samuels 2019).

p110α is a widely expressed PI3K isoform in vivo and a key intermediate in insulin-like growth factor-1 (IGF-1), insulin and leptin signalling, where it plays a key role in growth factor and metabolic signalling through highly selective recruitment and activation of the insulin receptor substrate (IRS) signalling complex (Hopkins et al. 2018). p110α is significantly expressed in endothelial cells and its activity is necessary for vascular development. Severe defects in angiogenic sprouting and vascular remodelling caused by generalized or endothelial cell-specific inactivation of p110α lead to embryo death in the second trimester (Araiz et al. 2019).

### Alpelisib

Alpelisib (Piqray™; BYL719; Novartis) is an oral, highly selective inhibitor of PI3Kα, that received approval on May 2019 by the FDA and is indicated in combination with fulvestrant for the treatment of postmenopausal women, and men, with HR-positive, HER2-negative, *PIK3CA*-mutated, advanced or metastatic breast cancer (Markham 2019). The SOLAR-1 trial (NCT02437318) showed that the PFS of breast cancer patients treated with alpelisib plus fulvestrant was significantly longer than that of patients treated with placebo-fulvestrant (11 months versus 5.7 months), and the overall response rate was greater (26.6% versus 12.8%) (Andre et al. 2019). Furthermore, in a phase Ib clinical trial (NCT01623349), alpelisib in combination with olaparib was also tolerable and effective in patients with triple-negative breast cancer. The median OS of the enrolled patients was 11.8 months (Batalini et al. 2022).

Given the effectiveness of alpelisib, other combined strategies are still in development, and toxicity management should be considered simultaneously (Rugo et al. 2021). In a first-in-human study (NCT01219699) with 134 patients with *PIK3CA*-mutated solid tumours, 13.4% of patients discontinued treatment because of serious adverse events, but overall, the safety was tolerable (Juric et al. 2018). Hyperglycaemia, rash and diarrhoea occurred frequently during alpelisib treatment, among which only diarrhoea occurred later (Andre et al. 2021; Rugo et al. 2020). Symptomatic treatment can alleviate adverse reactions to a certain extent. Hyperglycaemia can be relieved with metformin; receiving H1 antihistamines can prevent rash; notably, grade ≥ 3 diarrhoea occurs in only 7% of patients and often does not require intentional intervention (Sharma et al. 2021).

### PI3Kβ inhibitors

P110β regulates insulin metabolism, cell proliferation and trafficking in a kinase-independent way, and conditional knockout of p110β in mouse liver results in impaired insulin sensitivity and glucose homeostasis with little effect on protein kinase B (Akt) phosphorylation (Fujikawa et al. 2019; Zhang et al. 2021). The development of *PTEN*-deficient tumours is dependent on p110β signalling. p110β is a crucial kinase driving the survival of *PTEN*-deficient cancer cell lines in vitro (Zhang et al. 2017). In vivo, knockdown of p110β inhibited tumour formation in a mouse model of *PTEN*-deficient prostate tumours (Mao et al. 2021). However, recent studies have shown that *PTEN*-deficient breast cancer cell lines, which are resistant to PI3K inhibitors, have a highly activated p110β D1067Y mutant and induce elevated phosphatidylinositol 3,4,5-trisphosphate (PIP3) levels, resulting in hyperactivation of the PI3K pathway (Nakanishi et al. 2016). In other respects, the expression level of p110β is highly correlated with the high incidence and poor survival of glioblastoma (Pridham et al. 2018), and p110β inhibitor monotherapy or combined c-Jun N-terminal kinase (JNK) inhibitor can significantly inhibit the growth of xenograft tumours (Zhao et al. 2016).

### AZD8186

AZD8186 (AstraZeneca) is a potent and selective inhibitor of PI3Kβ and PI3Kδ (Barlaam et al. 2015). Preclinically, AZD8186 has fairish activity across tumour cells and *PTEN*-null tumour models (Lynch et al. 2017) alone or in combination with docetaxel (Hancox et al. 2015), androgen deprivation (Marques et al. 2015), PI3Kα inhibitor (Schwartz et al. 2015) and mitogen-activated protein kinase kinase (MEK) inhibitor (Marques et al. 2020). The first-in-human, phase I study (NCT01884285) characterized the favourable safety and tolerability of AZD8186 in patients with advanced solid tumours, and in the present study, the most common adverse events were diarrhoea, nausea and vomiting (Choudhury et al. 2022).

### GSK2636771

GSK2636771 (GlaxoSmithKline) is an orally bioavailable, selective inhibitor of PI3Kβ. As with AZD8186, the first-in-human clinical trial (NCT02215096) results confirmed promising clinical activity as a single agent and manageable toxicity profile of this PI3Kβ inhibitor (Mateo et al. 2017). Even so, no further clinical trials have shown its effectiveness. Although GSK2636771 plus enzalutamide was tolerated in patients with metastatic castration-resistant prostate cancer, it still has limited antitumour activity and needs other combinations to achieve clinical benefit (Sarker et al. 2021). The most common adverse events related to GSK2636771 included diarrhoea, nausea and vomiting (Mateo et al. 2017).

### PI3Kδ inhibitors

P110δ is mainly expressed in leukocytes and exists as an oncogenic driver in various solid tumours, such as breast cancer, prostate cancer and colorectal cancer (Xenou and Papakonstanti 2020). Although no mutation of p110δ was detected in breast cancer, its expression level gradually increased during the progression of human breast cancer (Goulielmaki et al. 2018). Inactivation of p110δ in macrophages inhibits mammary tumour growth and reduces the recruitment of macrophages in mice, and PI3Kδ inhibitors may be considered in clinical trials for the treatment of breast cancer in the future (Goulielmaki et al. 2018). Therefore, many studies on p110δ-selective inhibitors as monotherapy or combination therapy to improve cancer immunotherapy are being actively carried out.

p110δ inhibition has significant immunomodulatory activity, causing immune-mediated adverse effects by preferentially inhibiting regulatory T cells (Tregs). An intermittent dosing regimen alleviated adverse effects while maintaining the antitumour activity of PI3Kδ inhibitors in a phase II trial. Moreover, the intermittent dosing regimen in mouse models of melanoma resulted in a marked reduction in tumour growth without inducing pathogenic T cells in colon tissue (Eschweiler et al. 2022).

### Idelalisib

Idelalisib (Zydelig™; CAL-101; Gilead Sciences) is a first-in-class, highly specific, small molecule inhibitor targeting p110δ, that was indicated, in combination with rituximab, for the treatment of patients with relapsed chronic lymphocytic leukaemia (CLL) by the FDA in 2014. The drug has also been approved in patients with relapsed follicular B cell non-Hodgkin lymphoma and relapsed small lymphocytic lymphoma (SLL) who have received at least two prior systemic therapies (Markham 2014; Yang et al. 2015). However, this approval also carries a black box warning: fatal and serious toxicity related to idelalisib includes hepatic toxicity, severe diarrhoea, colitis, pneumonitis, infections and intestinal perforation. Therefore, the FDA withdrew accelerated approval of idelalisib for both FL and SLL on Feb. 18, 2022. In a phase III study (NCT01539512), the combination of idelalisib and rituximab significantly improved OS at 12 months (92% in the idelalisib group versus 80% in the placebo group), with an acceptable incidence of adverse events due to the short follow-up in this study (Furman et al. 2014). In another phase III study (NCT01539291), the longer-term data showed that diarrhoea increased with longer exposure, while the incidence of elevated transaminases plateaued. Most patients discontinued treatment during the trial due to serious adverse events (Sharman et al. 2019). As the first approved PI3K inhibitor, many preclinical studies on idelalisib are still ongoing to address the high incidence of adverse events. Recent reports imply that idelalisib exposure deeply impairs human dendritic cell differentiation and function in vitro, which partly explains the mechanisms of idelalisib-associated infections (Braun et al. 2021). In addition, idelalisib might increase the risk of infections in all B cell malignancies by inducing dysfunction of natural killer cells and T cells (Rohrbacher et al. 2021). On-target toxicity is the major limitation of idelalisib. Given the patients who are refractory to first-line therapy, further clinical trials are needed to develop a new combination of approaches to reduce toxicities (Tomowiak et al. 2021). Disappointingly, while combining idelalisib with Bruton's tyrosine kinase (BTK) inhibitors at a lower dose is overall well tolerated, there no sufficient efficacy advantage was obtained (Morschhauser et al. 2021).

### Duvelisib

Duvelisib (Copiktra^™^; IPI-145; Verastem) is a selective dual inhibitor of PI3Kδ and PI3Kγ, that received approval by the FDA for the treatment of adult patients with relapsed or refractory CLL and SLL and accelerated approval for FL after at least two prior therapies in 2018 (Blair 2018). Duvelisib has shown meaningful efficacy and acceptable safety as monotherapy for CLL/SLL patients (Flinn et al. 2018). Studies are ongoing about combination treatment of duvelisib or its activity in advanced haematologic malignancies. In patients with relapsed/refractory CLL, combination therapy with rituximab did not result in new toxicities, but its PFS (13.7 months) did not significantly outperform duvelisib alone (PFS, 13.3 months) (Davids et al. 2021a; Flinn et al. 2019a). However, inhibition of PI3K reversed resistance to BTK inhibitors in mantle cell lymphoma (Zhao et al. 2017). Treatment with duvelisib and the B cell lymphoma-2 (Bcl-2) inhibitor venetoclax synergistically induces tumour regression in Richter syndrome patient-derived xenograft (PDX) models (Iannello et al. 2021). In a DYNAMO study (NCT01882803), duvelisib monotherapy had to encourage clinical activity in patients with refractory indolent non-Hodgkin lymphoma; the median PFS was 10 months, yet 31% of patients required treatment discontinuation as immune-related toxicities (Flinn et al. 2019b). Duvelisib carries a black box warning for the risk of fatal and/or serious infections, diarrhoea or colitis, cutaneous reactions and pneumonitis. On Oct. 17, 2021, the FDA withdrew accelerated approval of duvelisib treatment for FL. Considering the high activity of duvelisib, many investigations are underway to relieve frequent adverse events, including diverse combinations of drugs and alternative dosing schedules.

### Umbralisib

Umbralisib (UKONIQ™; TGR-1202; TG Therapeutics) is a dual PI3Kδ and casein kinase 1 epsilon (CK1ε) inhibitor with accelerated approval by the FDA for the treatment of adult patients with relapsed or refractory marginal zone lymphoma (MZL) who have received at least one prior anti-CD20-based regimen and refractory FL who have received at least three prior lines of systemic therapy on 2021 (Dhillon and Keam 2021). At a median follow-up of 27.7 months, the objective response rates (ORRs) were 49.3 and 45.3% for patients with MZL and FL, respectively (NCT02793583). MZL did not reach the median PFS. For FL, the median PFS was 10.6 months (Fowler et al. 2021). Studies using it as combination therapy are still ongoing, a triplet approach in which umbralisib and ublituximab plus ibrutinib (NCT02006485) are well tolerated and effective in patients with CLL (Nastoupil et al. 2019; Roeker et al. 2022). In terms of clinical trial results, umbralisib has achieved encouraging clinical activity with a relatively low incidence of adverse events. The most common grade 3 or higher adverse events were neutropenia (8.9%), diarrhoea (6.7%) and increased aminotransferase levels (5.4%), which are lower than those of other PI3Kδ inhibitors (Davids et al. 2021b). It was reported that inhibition of CK1ε can preserve Tregs number and function, which may modulate immune-mediated adverse events (Maharaj et al. 2020). However, on May. 31, 2022, FDA withdrew accelerated approval of umbralisib for both MZL and FL due to increased risk of death for patients.

### PI3Kγ inhibitors

P110γ is highly expressed in myeloid cells, and inhibition of p110γ can reshape the tumour immune microenvironment and restore the sensitivity of tumours rich in tumour-associated myeloid cells to immune checkpoint inhibitors (De Henau et al. 2016). Knockout phosphatidylinositol-4,5-bisphosphate 3-kinase catalytic subunit gamma (*PIK3CG*) increased CD8^+^ T cell infiltration and programmed death 1 (PD-1) expression in T cells in the tumour microenvironment; thus, inhibiting p110γ enhanced the efficacy of anti-programmed cell death ligand 1 (PD-L1) immunotherapy in head and neck squamous cell carcinoma by modulating host immune activity against tumour cytotoxicity and increasing the expression of immunosuppressive markers (Anderson et al. 2021). In addition, activation of PI3K signalling cooperates with Kirsten rat sarcoma (KRAS) viral oncogene homologue to promote the development of aggressive pancreatic ductal adenocarcinoma in vivo, and knockdown of p110γ inhibits KRAS-induced tumour growth. However, in the context of a high-fat diet, knockdown of p110γ caused p-Akt activation and liver injury instead (Torres et al. 2019). On the other hand, targeting p110γ can interfere with doxorubicin-induced cardiotoxicity and synergistically enhance anticancer effects. p110γ-mediated blockade of autophagy drives doxorubicin-induced cardiotoxicity while blocking T-cell-mediated tumour killing by promoting trafficking and differentiation of immunosuppressive macrophage subsets (Li et al. 2018).

### IPI-549

IPI-549 (Infinity Pharmaceuticals), the only efficient and highly selective PI3Kγ inhibitor in clinical development, is currently being evaluated in some clinical trials. In vitro, targeting p110γ with IPI-549 can reshape the tumour immune microenvironment and overcome resistance to checkpoint blockade therapy in myeloid cells (De Henau et al. 2016). In vivo, IPI-549 remodels the suppressive tumour microenvironment alike by inhibiting p110γ in both murine pancreatic cancer and melanoma models (Zhang et al. 2019). Furthermore, the codelivery of IPI-549 and silibinin to breast tumours can act synergistically (Jiang et al. 2020). IPI-549 treatment mitigates abdominal aortic aneurysm formation in mice by inhibiting Akt phosphorylation (Liu et al. 2020). Even though there are still no successful PI3Kγ inhibitors recognized in the clinic, the advance of IPI-549 in preclinical research testifies to the viability and druggability of this kinase. Clinical trials of IPI-549 for triple-negative breast cancer (NCT03719326) and head and neck squamous cell carcinoma (NCT03795610) are ongoing.

### Mechanism and management of the most relevant toxicity

The FDA presented in a briefing at the oncologic drug advisory committee (ODAC) meeting in April 2022 that six randomized controlled trials of PI3K inhibitors for haematologic malignancies showed consistent results: in the context of PFS advantage or potential advantage, there has been a downwards trend in OS, and this trend may be due to toxicity. Clinical data from single-arm trials limit the interpretation of efficacy and safety. In the absence of a comparator arm, the observed side effects can be attributed to the drug or the underlying disease, making it difficult to determine which is responsible. At the same time, single-arm trials have relatively short follow-up periods, which limits confidence in assessing long-term safety (Meeting of the oncologic drugs advisory committee 2022).

Although the efficacy of PI3K inhibitors has been reported in a variety of tumours, and several inhibitors have been approved for marketing, the serious and even lethal toxicity they cause remains a problem that cannot be ignored (De Santis et al. 2019). In addition to playing various roles in tumour proliferation, different subunits of PI3K are often associated with multiple physiological functions, such as glucose metabolism, inflammation and immunity (Fruman et al. 2017). The dosage of drugs that can inhibit tumours is bound to affect the normal function of other cells, resulting in severe side effects (He et al. 2021). The following summarizes the serious toxicity caused by PI3K inhibitors in clinic (Table 3), including hyperglycaemia, cutaneous reactions, diarrhoea/colitis, pneumonitis, hepatotoxicity and hypertension.

**Table 3:** Serious adverse events (AEs) of FDA-approved PI3K inhibitors

FDA-approved drugs	Copanlisib (Dreyling et al. 2017a) (*n* = 84)	Alpelisib (Andre et al. 2019) (*n* = 284)	Idelalisib (Gopal et al. 2014) (*n* = 125)	Duvelisib (Flinn et al. 2018) (*n* = 160)	Umbralisib (Fowler et al. 2021) (*n* = 208)
Grade ≥ 3 AEs					
Pneumonitis	2%	1.1%	7%	14%	3%
Cutaneous reactions	0%	10%	2%	2%	2%
Diarrhoea/colitis	5%	7%	13%	15%/12%	10%
Hepatotoxicity	6%	–	21%	6%	7%
Hyperglycemia	25%	37%	–	–	–
Hypertension	41%	–	–	–	–
Discontinuation due to AEs	21%	25%	20%	35%	15%

### Hyperglycaemia

Pan-PI3K inhibitors and PI3Kα inhibitors usually generate severe hyperglycaemia, grade ≥ 3, which was experienced by 23.8% of patients treated with copanlisib (Dreyling et al. 2017a) and 32.7% of patients treated with alpelisib (Rugo et al. 2020). In a phase II study of copanlisib (NCT01660451) in patients with relapsed or refractory indolent lymphoma, the incidence of hyperglycaemia (grade ≥ 3) was up to 41% (Dreyling et al. 2017b).

Management of hyperglycaemia usually involves once/twice-daily glucose monitoring and treatment with oral antihyperglycaemic agents (metformin, first line) (Goldman et al. 2016). Hyperglycaemia typically occurs during the first two cycles of treatment with PI3K inhibitors (Andre et al. 2019; Lenz et al. 2020). Upon treatment initiation, patients should follow dietary guidelines. Once hyperglycaemia was detected, a mild increase was relieved by metformin 500 mg once daily. If symptoms cannot be improved, adding an insulin sensitizer, such as pioglitazone, can be considered. However, if hyperglycaemia is still serious despite treatment with antidiabetic medications, the PI3K inhibitor should be dose reduced or discontinued (Nunnery and Mayer 2019).

Hyperglycaemia is considered an on-target effect of PI3K inhibitors, which is relevant to the crucial role of the PI3K pathway in insulin signalling and glucose homeostasis (Juric et al. 2018). In addition, p110α and p110β modulate insulin-driven PI3K/Akt signalling, the inhibition of which would result in hyperglycaemia rather than p110δ and p110γ (Molinaro et al. 2019). Moreover, glucose-insulin feedback induced by PI3K inhibitors is sufficient to reactivate PI3K signalling and compromise their effectiveness (Hopkins et al. 2018).

### Cutaneous reactions

Cutaneous reactions (rash or maculopapular rash) are one of the most common toxicities with PI3K inhibitors in trials. A grade 3 or higher maculopapular rash was reported in 17% of patients treated with duvelisib (Horwitz et al. 2018) and 13% of patients treated with alpelisib (Juric et al. 2019). In the treatment of CLL with idelalisib, rash was reported in 10 to 22% of patients receiving monotherapy and 58% of patients using rituximab in combination (Huilaja et al. 2018). In particular, three patients with PI3K inhibitors developed diffuse erythroderma and keratoderma (Dewan et al. 2018).

Mild rash intervention with topical corticosteroid treatment and oral antihistamine as appropriate, systemic corticosteroids and discontinued PI3K inhibitors in more severe cases (Rugo et al. 2020). Moreover, topical or oral antibiotics, topical antipruritic agents and γ-aminobutyric acid agonists are also considered if symptoms cannot be controlled (Esposito et al. 2019).

PI3K/Akt signalling determines the choice between differentiation and death of epidermal keratinocytes (Li et al. 2019). Inhibition of this pathway also suppresses the proliferation and migration of keratinocytes (Wu et al. 2022). In addition, activation of the PI3K/Akt/mammalian target of rapamycin (mTOR) pathway inhibits autophagy and promotes inflammation in keratinocytes (Varshney and Saini 2018). Presumably, inhibition of PI3K is responsible for this toxicity. Unfortunately, current reports on the mechanism of cutaneous reactions remain limited.

### Diarrhoea/colitis

Common diarrhoea/colitis caused by PI3K inhibitors has been widely reported, even resulting in many patients discontinuing protocol therapy because of severe diarrhoea (Curigliano and Shah 2019). In 84 patients treated with copanlisib monotherapy, the incidence of diarrhoea was 40.5% (any grade) and 4.8% (grade ≥ 3) (Dreyling et al. 2017a); the diarrhoea of any grade that occurred in patients with alpelisib was 57.7% (grade ≥ 3, 6.7%) (Andre et al. 2019); and the most common adverse event related to idelalisib was colitis/diarrhoea (37%; grade ≥ 3, 15%) (Lampson et al. 2019). Colitis and diarrhoea were the only adverse events reported in 5% of the duvelisib-treated patients who discontinued treatment (Flinn et al. 2018). More seriously, idelalisib causes fatal intestinal perforation (Barrientos 2016). An expert panel identified two types of diarrhoea caused by idelalisib. The first type is self-limiting and generally occurs within the first 8 weeks, which is mild and responds to antidiarrhoeal agents. The second type of diarrhoea is considered most likely to be related to idelalisib; it tends to occur relatively late and does not respond well to antidiarrhoeal or empiric antimicrobial therapy (Coutre et al. 2015).

Initial management of diarrhoea induced by PI3K inhibitors should include evaluation and work-up to rule out infection, followed by treatment with oral or intravenous budesonide after exclusion of infection causes (Coutre et al. 2015). Additionally, patients could also be instructed to use loperamide followed by opium tincture and octreotide acetate, and diphenoxylate hydrochloride/atropine sulphate may be used in place of loperamide (Nunnery and Mayer 2019). Eventually, treatment with PI3K inhibitors should be reduced or even interrupted if patients experience unresolved diarrhoea (Esposito et al. 2019).

Histologically, colonoscopy examination in patients who had colitis found neutrophilic infiltration of the crypt epithelium, intraepithelial lymphocytosis and crypt cell apoptosis (Weidner et al. 2015). The increased intraepithelial lymphocytes were mostly CD8^+^ T cells, which may be caused by immune dysregulation (Louie et al. 2015). In mouse models, PI3Kδ knockout developed histologic findings similar to idelalisib-associated diarrhoea (Uno et al. 2010), and mesenteric B cells protect against mucosal injury by inhibiting Tregs. Therefore, PI3Kδ inhibitors may inhibit B cell differentiation through immune dysregulation of Tregs, leading to intestinal injury (Louie et al. 2015).

### Pneumonitis

Fatal and serious pneumonitis has occurred in patients treated with PI3K inhibitors. Copanlisib-related pneumonitis was reported in 21 of 307 (7%) patients and even led to death in 2% of patients (Matasar et al. 2021). Pneumonitis was the most commonly reported infectious adverse effect in duvelisib-treated patients (18%), and *Pneumocystis jirovecii* pneumonia (PJP) occurred in three patients (Flinn et al. 2018). Similarly, there were four occurrences of PJP infections in patients treated with idelalisib (Sharman et al. 2019). The clinical features of idelalisib-related pneumonitis include cough, dyspnoea and fever, and lung computed tomography scans showed diffuse ground-glass opacities, consolidations, diffuse micronodules and pleural effusions (Haustraete et al. 2016).

The United States prescribing information for idelalisib recommends that patients who are treated with PI3K inhibitors should be monitored for respiratory symptoms, such as cough, dyspnoea, hypoxia, interstitial infiltrates on a radiologic examination, or oxygen saturation drops more than 5% (Coutre et al. 2015). PI3K inhibitors should be immediately interrupted until the cause of pneumonitis has been determined. A subsequent infectious aetiological evaluation for pneumonia should be performed (Esposito et al. 2019). Patients may require oxygen supplementation and have bronchoalveolar lavage. However, broad-spectrum antibiotic therapy has little effect on such patients. Treatment with systemic corticosteroids and drug withdrawal present a favourable outcome (Haustraete et al. 2016).

Currently, there is no in-depth report on the pathogenesis of PI3K inhibitor-related pneumonia. Noninfectious pneumonitis may be related to the inhibition of PI3K downstream. PI3K inhibitors also inhibit the mTOR pathway by inhibiting PI3K. Both hypersensitivity pneumonitis and organizing pneumonia have occurred in patients treated with mTOR inhibitors (Albiges et al. 2012). Pneumonitis is also an immunologic disorder. Median increments in cytokines/chemokines associated with immune cell recruitment and T-helper type 1 (Th1) responses, including interferon-γ and interleukins 6, 7, and 8, were observed in serum samples from patients with idelalisib-related pneumonia (Barr et al. 2016).

### Hepatotoxicity

Severe hepatotoxicity is an idelalisib-specific adverse effect that is not shared with the other PI3K inhibitors. Although transaminitis occurred in copanlisib treatment, elevations in ALT and AST were generally less severe (Matasar et al. 2021). Patients treated with idelalisib experienced elevated ALT levels (39.1%) and AST levels (28.2%) during the primary study which was not associated with prolonged drug exposure (Sharman et al. 2019). Elevations in ALT or AST more than five times the upper limit of normal (ULN) were observed within the first 12 weeks of treatment, most of which were reversible with dose interruption (Coutre et al. 2015). Idelalisib plus the immunomodulator lenalidomide significantly increased the elevation of transaminase, leading to death in two cases (Lampson and Brown 2017). In contrast, younger patients with immunoglobulin heavy chain variable region (*IGHV*)-mutated disease tend to be more likely to develop early hepatotoxicity caused by idelalisib (Lampson et al. 2016).

Hepatic function monitoring should be performed every 2 weeks for the first 3 months of treatment to prevent transaminitis; drugs should be temporarily discontinued when transaminases are increased over 5 times the ULN (grade 3); steroid-based treatment should be given if transaminitis does not significantly decline after 7 days of the drug withdrawal; and elevations in transaminases more than 20 times over the ULN (grade 4) should permanently discontinue the drug (Cuneo et al. 2019; Nair and Cheson 2016).

Idelalisib-related hepatotoxicity is immune mediated, the cause of which is irrelevant to the virus (Lampson et al. 2016). Liver biopsy showed increased infiltration and activation of CD8^+^ T cells. Cytokine analysis found that the serum levels of the proinflammatory cytokines C–C motif chemokine ligand (CCL)-3 and CCL-4 were significantly upregulated. Moreover, patients who experience hepatotoxicity usually have a visibly reduced Tregs population, 68% of which lost 42% of the Tregs fraction (Lampson et al. 2016).

### Hypertension

Hypertension is one of the most common adverse events, occurring in 49% of patients treated with copanlisib plus rituximab (Matasar et al. 2021) and 40.3% of patients treated with monotherapy (Lenz et al. 2020). Hypertension reported with long-term exposure to copanlisib was transient and manageable. The incidence of grade 3 was essentially unchanged (23.9%) compared to the primary study (23.2%) (Dreyling et al. 2020). Blood pressure peaked 1–2 h after transfusion and then declined to baseline levels within 24 h (Dreyling et al. 2017a). The mean changes in systolic and diastolic blood pressure during this period were 16.8 mmHg and 7.8 mmHg, respectively (Cheson et al. 2019). Few patients discontinued due to severe hypertension, and short-acting antihypertensive drugs nifedipine or reducing the dose of copanlisib are generally chosen when blood pressure cannot be controlled (Cheson et al. 2019).

Although the specific mechanism of copanlisib-related hypertension has not been identified thus far, it may be associated with acute vasoconstriction because of the mode of intravenous infusion (Lenz et al. 2020; Matasar et al. 2021). The PI3K/Akt pathway is involved in the regulation of canonical endothelial functions such as the regulation of vascular tone and leukocyte recruitment to the vessel wall (Mishra et al. 2021). As one of the targets of copanlisib, p110γ is a key factor that affects blood pressure levels. On one hand, p110γ relieves hypertension and reduces the inflammatory response in vascular tissue by reducing peripheral resistance; on the other hand, it may play a decisive role in the occurrence of hypertension and related target organ damage by regulating the function of T cells (Perrotta et al. 2016).

## Conclusion

There is no doubt that the activation of the PI3K pathway is crucial to the occurrence and development of tumours (Janku et al. 2018). This pathway is widely dysregulated in a variety of human cancers, including haematological malignancies, breast cancer and colorectal cancer, illustrating the potential value of developing PI3K inhibitors (De Santis et al. 2019). Currently, FDA-approved inhibitors include the pan-PI3K inhibitor copanlisib, PI3Kα inhibitor alpelisib, PI3Kγ/δ inhibitor duvelisib, PI3Kδ inhibitor idelalisib and umbralisib. In addition to alpelisib for the treatment of breast cancer, the approved indications for other inhibitors are haematological malignancies. Meanwhile, exploring the application of PI3K inhibitors in solid tumours has become a new hotspot.

However, with the launch of PI3K inhibitors, serious safety issues related to p110δ are increasingly exposed (Curigliano and Shah 2019). p110δ is preferentially expressed mainly in the haematopoietic system and affects immune cell development and function (Braun et al. 2021). On one hand, PI3Kδ inhibitors, thus, show significant efficacy in the treatment of haematological malignancies due to the regulation of immune cells (Xenou and Papakonstanti 2020). On the other hand, the immune-activating effects of PI3Kδ inhibitors have resulted in severe and lethal immune-related adverse reactions, such as hepatotoxicity, pneumonia, colitis and even intestinal perforation (Roskoski 2021). Furthermore, regulation of insulin signalling by p110α also contributes to PI3Kα inhibitor-related hyperglycaemia (Molinaro et al. 2019).

The PI3K inhibitors approved by the FDA based on single-arm trials have basically failed to show their due advantages. Although single-arm trials allow the evaluation of ORRs, they cannot accurately assess PFS and OS, and it is difficult to accurately characterize the efficacy and toxicity observed in patients. Approval of PI3K inhibitors will be significantly more difficult in the future due to serious safety issues. There are three recommendations made by ODAC: first, advocate careful dose selection through robust dose exploration in early randomized trials; second, avoid single-arm trials as a regulatory strategy in favour of randomized trials; third, comprehensively collect and analyse OS data to assess the effect of the drug on this “ultimate safety endpoint”. In brief, the above three points are achieved to determine a real and credible safety window of PI3K inhibitors, thereby ensuring the efficacy and safety of treatment.

Overall, first, on-target toxicities severely limit the development of PI3K inhibitors, and understanding the physiological functions of different isoforms of PI3K and their distribution in tissues is helpful for the clinical prediction of adverse effects. In addition to the need for corresponding adverse event management guidelines, it is also necessary to further clarify the mechanism of toxicities and find targeted intervention strategies. In addition, the side effects of PI3K inhibitors are closely related to their high-dose clinical application. Therefore, a large number of clinical studies are needed to determine the balance point of immunoregulation with PI3K inhibitors and to reduce side effects by optimizing low-dose and multiple administration. Future development of PI3K inhibitors requires exploring new intermittent delivery modalities or combination regimens to reduce the clinical dose of PI3K inhibitors while ensuring antitumour efficacy. Second, the lack of biomarkers also limits the clinical application of PI3K inhibitors. For example, stratification of breast tumours according to single and multiple copies of *PIK3CA* mutations resulted in distinct distributions of scores for PI3K signalling and cellular stemness (Madsen et al. 2021). This suggests that more genomic studies are still needed to accurately assess patient stratification for PI3K-targeted therapy and to identify a biomarker that can effectively predict patient susceptibility to PI3K inhibitors.

## Data Availability

The authors confirm that all the data are available within the text of the review.

## Abbreviations

Akt: Protein kinase B
ALT: Alanine aminotransferase
AST: Aspartate aminotransferase
Bcl-2: B cell lymphoma-2
BTK: Bruton's tyrosine kinase
CCL: C–C motif chemokine ligand
CK1ε: Casein kinase 1 epsilon
CLL: Chronic lymphocytic leukaemia
FDA: Food and Drug Administration
FL: Follicular lymphoma
GPCR: G-protein-coupled receptor
HR: Hormone receptor
IGF-1: Insulin-like growth factor-1
IRS: Insulin receptor substrate
JNK: C-Jun N-terminal kinase
KRAS: Kirsten rat sarcoma
MEK: Mitogen-activated protein kinase kinase
HER2: Human epidermal growth factor receptor 2
ODAC: Oncologic drug advisory committee
PIP2: Phosphatidylinositol 4,5‑bisphosphate
PIP3: Phosphatidylinositol 3,4,5-trisphosphate
*PTEN*: Phosphatase and tensin homolog
*PIK3CA*: Phosphatidylinositol-4,5-bisphosphate 3-kinase catalytic subunit alpha
*PIK3R1*: Phosphoinositide-3-kinase regulatory subunit 1
*PIK3CG*: Phosphatidylinositol-4,5-bisphosphate 3-kinase catalytic subunit gamma
*IGHV*: Immunoglobulin heavy chain variable region
mTOR: Mammalian target of rapamycin
MZL: Marginal zone lymphoma
ORRs: Objective response rates
OS: Overall survival
PD-1: Programmed death 1
PD-L1: Programmed cell death ligand 1
PDX: Patient-derived xenograft
PFS: Progression-free survival
PI3K: Phosphatidylinositol 3-kinase
PJP: *Pneumocystis jirovecii* Pneumonia
RTK: Receptor tyrosine kinase
SLL: Small lymphocytic lymphoma
Th1: T-helper type 1
Tregs: Regulatory T cells
ULN: Upper limit of normal
